# Reevaluating Calf Circumference as an Indicator of Muscle Mass in Malnutrition Among Community-Dwelling Older Adults: A Cross-Sectional Analysis

**DOI:** 10.3390/geriatrics10060162

**Published:** 2025-12-05

**Authors:** Emanuele Marzetti, Hélio José Coelho-Júnior

**Affiliations:** 1 Fondazione Policlinico Universitario Agostino Gemelli IRCCS, L.go A. Gemelli 8, 00168 Rome, Italy; 2 Department of Geriatrics, Orthopedics and Rheumatology, Università Cattolica del Sacro Cuore, L.go F. Vito 1, 00168 Rome, Italy

**Keywords:** muscle atrophy, malnourished, anorexia, appendicular skeletal muscle, DEXA

## Abstract

**Aim:** The present study aimed to evaluate the agreement between calf circumference (CC) and dual-energy X-ray absorptiometry (DEXA) in assessing muscle mass, and to determine how possible discrepancies influence the diagnosis of malnutrition and its relationship with frailty and disability in older adults. **Methods:** We analyzed cross-sectional data from 1048 adults aged 65 years and older who participated in the 2001–2002 National Health and Nutrition Examination Survey (NHANES). Malnutrition was defined using the Global Leadership Initiative on Malnutrition (GLIM) criteria, and muscle mass was estimated using both DEXA and CC. Agreement between the two assessment methods was tested with Kappa statistics, while multivariable logistic regression models were used to explore the associations between malnutrition (as determined by each method) and frailty or disability, controlling for age, sex, physical activity, polypharmacy, and urinary albumin levels. **Results:** CC and DEXA-based appendicular skeletal muscle mass (ASM) showed a moderate correlation (r = 0.592). The prevalence of malnutrition was 10.3% when defined by CC and 9.1% when defined by DEXA (κ = 0.635, *p* = 0.001). In both cases, malnutrition was significantly associated with frailty (OR: 1.56; 95% CI: 1.240, 1.970, *p* < 0.001), but not with disability. Adjusting for albumin levels did not substantially change these associations. **Conclusions:** CC and DEXA demonstrate moderate concordance in estimating ASM. While this level of agreement slightly affects malnutrition prevalence estimates, it does not alter the observed relationship between malnutrition and frailty or disability in older adults.

## 1. Introduction

Malnutrition is a condition resulting from inadequate nutrient intake or absorption, which leads to significant alterations in body composition and impaired physiological functions [1]. It is especially common among older adults and has garnered significant attention from global health organizations due to its potential to increase the risk of adverse health outcomes as it progresses [2,3,4].

However, this understanding has recently been questioned by studies showing mixed or inconsistent results [5]. One factor contributing to these discrepancies may be the different tools and criteria used to assess malnutrition across studies. To address this variability, the Global Leadership Initiative on Malnutrition (GLIM) [1] convened leading clinical nutrition bodies to develop a standardized approach for defining and diagnosing malnutrition. Since the introduction of this framework, a growing number of studies have explored the relationship between GLIM-defined malnutrition and various health outcomes [6].

The assessment of muscle mass is a key component within the GLIM criteria [1], which endorses the use of advanced imaging techniques (dual-energy X-ray absorptiometry (DEXA)), or alternative methods (e.g., calf circumference (CC)), when these instruments are not available [7]. However, CC and DEXA may not serve as equivalent measures of muscle mass. Indeed, some research has reported only moderate correlations between CC and DEXA in older adult populations [8,9,10,11]. Despite this, there is limited evidence on how these differences affect malnutrition prevalence estimates and their relationships with health outcomes.

Serum albumin is frequently measured in clinical settings due to its role as the primary plasma protein responsible for maintaining oncotic pressure [12,13]. Reductions in serum albumin can lead to decreased oncotic pressure and subsequent edema [12,13]. Yet, to our knowledge, no prior research has examined the potential moderating influence of albumin levels on the association between CC and malnutrition diagnosis.

Based on these premises, the current study examined the agreement between CC and DEXA in assessing muscle mass. Furthermore, we investigated how discrepancies between these measures influence malnutrition diagnosis and its association with health conditions. Finally, we explored the moderating effect of urinary albumin on the relationship between CC and malnutrition.

## 2. Materials and Methods

### 2.1. Study Participants

Data from the second cycle of the National Health and Nutrition Examination Survey (NHANES 2001–2002) were analyzed. NHANES has been designed to assess the health and nutritional status of the civilian, non-institutionalized population in the United States. Participants were selected through a complex, multistage probability sampling method [14]. The survey consists of interviews covering demographic, socioeconomic, dietary, and health-related information, as well as physical examinations that include medical measurements and laboratory tests [14]. Blood samples were obtained by trained and certified medical personnel at mobile examination centers [15]. The study protocol was approved by the Ethics Review Board of the National Center for Health Statistics (NCHS ERB), which ensures the protection of participants’ rights and adherence to U.S. federal regulations. Data collection for this research complied with NCHS ERB Protocol #98-12.

For the present analysis, older adults aged 65 years and above from the NHANES 2001–2002 cohort with complete data on both CC and DEXA were included (*n* = 1048).

### 2.2. Malnutrition

Malnutrition was operationalized according to the presence of at least one phenotypic and at least one etiologic criterion, as recommended by the GLIM criteria [1]. Phenotypic criteria included the following parameters: (a) unintentional weight loss ≥ 10% in the last 180 days; (b) low body mass index (BMI, <22 kg/m^2^); and (c) low muscle mass (appendicular skeletal muscle (ASM) < 20 kg for men and <15 kg for women) [16]. Etiologic criteria included: (a) reduced food intake (answered “Yes” to the question “During that two-week period (when the participants felt irritable), did you have less appetite than usual almost every day?”); and (b) inflammation, according to the presence of multimorbidity (≥3 chronic conditions).

Appendicular skeletal muscle (ASM) was estimated using two methods. CC was measured with a steel measuring tape while the participant was seated. The maximum circumference was recorded at a perpendicular plane to the long axis of the right calf, to the nearest 0.1 cm. Body composition was assessed at the Mobile Examination Center using a Hologic QDR 4500A fan-beam X-ray bone densitometer (Hologic Inc., Marlborough, MA, USA) with the Hologic Discovery software, version 12.1. Whole-body scans were conducted under standard conditions, with further details provided in the NHANES procedures manual [17]. Exclusion criteria for the DXA scan included participants weighing more than 136 kg (300 lbs), taller than 1.96 m (6 ft 5 in), or having undergone contrast-based radiological examinations within 72 h. Appendicular Lean Soft Tissue (ALST) was calculated as the sum of lean soft tissue from the legs and arms, then normalized for height squared to obtain the appendicular lean mass index (ALMI, expressed in kg/m^2^) [18].

### 2.3. Health Conditions

#### 2.3.1. Frailty

The presence of frailty was assessed using the FRAIL scale [19], which consists of five domains that evaluate key aspects of frailty. (1) Fatigue: Participants were asked, “Over the last 2 weeks, how often have you been bothered by feeling tired or having little energy?” Those who answered “several days”, “more than half the days”, or “nearly every day” received a score of 1 for this domain. (2) Resistance: Participants were asked, “By yourself and without using any special equipment, how much difficulty do you have lifting or carrying something as heavy as 10 pounds (like a sack of potatoes or rice)?”. A response of “No difficulty” was scored as 0, while “Some difficulty” or “Unable to do” received a score of 1. (3) Ambulation: Participants were asked, “By yourself and without using any special equipment, how much difficulty do you have walking for a quarter of a mile (about 2–3 blocks)?”. A response of “No difficulty” was scored as 0, and “Some difficulty” or “Unable to walk this distance” was scored as 1. (4) Illnesses: The number of chronic conditions diagnosed was recorded. A score of 1 point was assigned for each chronic condition, and a higher score indicated the presence of more health conditions. (5) Loss of Weight: Self-reported unintentional weight loss of 5% or more over the past year was assessed. If the participant reported this weight loss, 1 point was assigned for this domain. Each domain was scored dichotomously (0 = no issue, 1 = issue present). The total FRAIL score ranges from 0 to 5, with those with ≥3 points being classified as frail.

#### 2.3.2. Disability

Functional disability was assessed using a 19-item physical functioning questionnaire, covering five domains: (1) Activities of daily living (ADLs) (e.g., getting in and out of bed, dressing); (2) Instrumental activities of daily living (IADLs) (e.g., managing money, preparing meals); (3) Leisure and social activities (LSA) (e.g., attending social events, going to the movies); (4) Lower extremity mobility (LEM) (e.g., walking a quarter mile, climbing stairs); and (5) General physical activity (GPA) (e.g., lifting heavy objects, standing for long periods) [20]. Participants were considered to have a functional disability if they reported “some difficulty” or more in any item of a given domain.

### 2.4. Covariates

Age, sex, polypharmacy, and physical activity level were obtained via questionnaires, which were administered by trained interviewers. Physical activity (yes/no) was defined as active (moderate (at least 10 min per episode, only slight sweating and slight to moderate increase in breathing and heart rate) or vigorous activity (at least 10 min per episode, heavy sweating, and large increase in breathing and heart rate) in past 30 days) or inactive (no moderate or vigorous activity in past 30 days). Polypharmacy was defined as the use of five or more medications [21,22].

### 2.5. Statistical Analysis

Continuous variables are presented as means ± standard deviations (SDs), while categorical and ordinal variables are reported as absolute numbers and percentages. Associations between CC and DEXA-based ASM were examined using Pearson’s correlation. Kappa statistics were conducted to examine the agreement between the two measurement methods for malnutrition assessment. Unadjusted and adjusted logistic regressions were performed to examine the associations between malnutrition and both frailty and disability. All models were adjusted for age, sex, physical activity levels and polypharmacy. A significance level of 5% (*p*  <  0.05) was adopted for all statistical tests, which were two-tailed. Statistical analyses were conducted using SPSS software (version 23.0; SPSS Inc., Chicago, IL, USA).

## 3. Results

### 3.1. Participant Characteristics

The main characteristics of study participants (*n* = 1048) are shown in Table 1. Mean age (74.4 ± 6.3 years) and BMI values (27.6 ± 5.3 kg/m^2^) indicate they were relatively young older adults with overweight. Sex distribution was nearly balanced, with women slightly outnumbering men (50.9%). Average protein intake (0.89 ± 0.43 g/kg/day) was higher than the current recommended daily allowance (RDA), while mean caloric intake (1693.6 ± 689.9 kcal/day) was likely within the estimated requirements calculated according to the ESPEN guidelines (~1500–1875 kcal/day) [23].

### 3.2. Agreement for Malnutrition Between Assessment Methods

**Figure 1:**
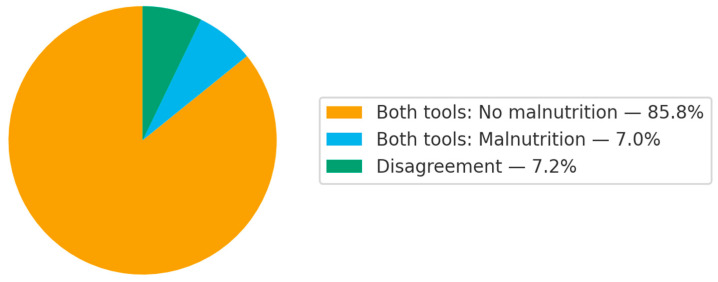
Agreement in malnutrition diagnosis using each assessment tool.

Figure 1 illustrates the prevalence of malnutrition according to assessment tools. The prevalence of malnutrition was 10.3% and 9.1% for CC and DEXA, respectively (κ = 0.635, 95% CI = 0.555, 0.715, *p*-value = 0.001).

### 3.3. Associations Between Assessment Methods

Table 2 shows the associations between assessment methods. CC (r = 0.592) and ASM derived from CC (r = 0.598) showed moderate correlation with DEXA-based ASM.

### 3.4. Associations Between Malnutrition with Disability and Frailty

**Figure 2:**
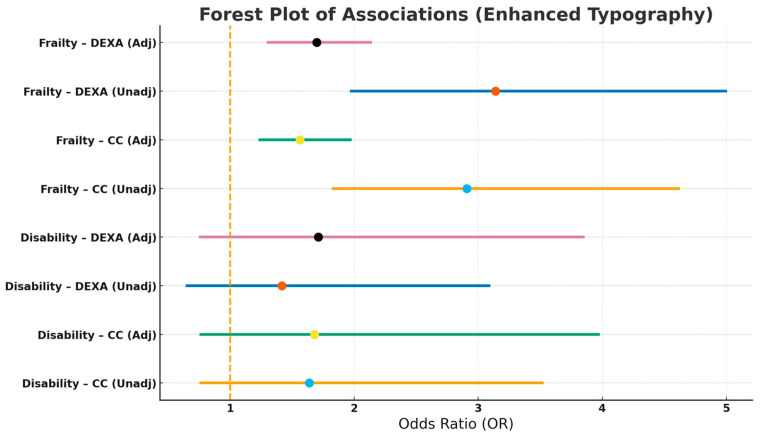
Associations Between Malnutrition with Disability and Frailty.

Table 3 and Figure 2 show the associations between malnutrition with disability and frailty. Malnutrition was significantly associated with frailty, whereas no significant association was observed with disability, regardless of the method used for estimating ASM. Further adjustments for albumin concentrations did not change the results.

## 4. Discussion

The main findings of the present study indicate that CC and DEXA are only moderately correlated among community-dwelling older adults. This scenario impacts the prevalence of malnutrition identified according to these assessment tools. However, no differences in the association with frailty and disability were found, even after adjustment for albumin levels.

Results of the present study are consistent with previous observations showing low-to-moderate correlations between CC and DEXA. Kiss et al. [8] found that only 60% of the variations in ASM index, estimated according to DEXA, were explained by CC among hospitalized older adults. In addition, authors observed that the optimal cutoff value for CC, defined using ROC curves, had modest capacity to identify individuals with low ASM index. Rolland et al. [9] found a moderate (r = 0.63) correlation between CC and DEXA-based ASM after examining community-dwelling French older adults who lived independently and had no history of mobility limitations. When researchers investigated the question of whether CC was a good discriminator of sarcopenia, the results indicated that it had a sensitivity of 44.3% and a specificity of 91.4%. Comparable associations between DEXA and CC were reported in studies that analyzed Asian cohorts [10,11], although slightly higher results for sensitivity and specificity were observed.

However, to best of our knowledge, this is the first study that examined if these differences could impact the prevalence of chronic conditions associated with muscle atrophy. Low muscle mass is a main determinant of malnutrition. As a matter of fact, the GLIM endorses low muscle mass as a phenotypic marker of malnutrition, with observational studies highlighting it as a predominant indicator among malnourished patients [24,25,26].

Collectively, these findings suggest that CC should not be used as an alternative to more precise assessment tools (e.g., DEXA) for estimating muscle mass in older adults. The moderate correlation between CC and DEXA, together with the modest agreement in malnutrition classifications derived from each method, supports this conclusion and highlights the possibility of false positives, as indicated by studies reporting low sensitivity. These considerations are particularly relevant in clinical settings, where small differences in CC values may lead to different diagnoses and therapeutic choices (e.g., malnutrition and supplementation use). Although it might be argued that CC could provide estimates of muscle mass in resource-limited settings, overall findings suggest that this approach could provide limited accuracy and potentially misleading classifications, thereby reducing its suitability even in such contexts.

In contrast, the use of this assessment tool in large-scale screening investigations might be considered. Indeed, the development of easy-to-apply and low-cost tools to assess muscle mass could enable the screening of large populations, the identification of individuals at higher risk, and the formulation of tailored public-health strategies. Nevertheless, a thorough review of the current CC-based equations for estimating muscle mass appears necessary to ensure more reliable results. In particular, the accuracy of CC-derived estimates is notably influenced by peripheral edema, varicose veins, obesity, among other alterations in body composition, which may compromise their validity in both research and practice. Future studies aimed at overcoming these limitations are urgently needed.

We found comparable associations between malnutrition and both disability and frailty, regardless of the method used to assess muscle mass. These observations could challenge the view that these tools are not similar proxies of muscle mass. However, these results should be interpreted with caution given multiple factors.

For instance, the findings of the present study are based on a cross-sectional design, which precludes conclusions about causality. Indeed, examining changes in frailty status over time may provide more meaningful insights, particularly in individuals who are already frail at baseline [27]. Furthermore, the possibility that results are a product of the combination of diagnostic criteria cannot be ruled out, as evidence has shown that other diagnostic criteria (e.g., weight loss and inflammation) may have a greater impact on the occurrence of adverse outcomes than low muscle mass [28].

We tested the possibility that albumin concentrations could impact associations with CC, but not with DEXA. This premise is grounded on the known impact of albumin on edema. Albumin refers to the most abundant protein [13]. This molecule is produced by hepatocytes and exerts a wide range of effects in the biological system, including the transport of substances (e.g., hormones, micronutrients, drugs), maintenance of blood volume, buffering of pH, and modulation of immune responses [13]. Nevertheless, a major function of albumin is the regulation of plasma oncotic pressure [12,13].

Under physiological conditions, albumin plays a key role in maintaining fluid balance by drawing water back into the capillaries from the surrounding tissues [13]. This action counterbalances hydrostatic pressure, which tends to push fluid out of the capillaries, thereby ensuring proper fluid distribution within the circulatory system and preventing excessive fluid accumulation in the interstitial spaces [12,13]. However, when albumin levels decrease, in response to various conditions (e.g., liver disease, malnutrition, or inflammation), the oncotic pressure is reduced, promoting the leakage of fluid into the interstitial space, which ultimately results in edema [12,13].

The lack of significant findings in the present study may be partly explained by our reliance on urinary rather than serum albumin, due to the limited availability of serum measures in NHANES. This constitutes a major limitation, as urinary albumin is not a valid indicator of protein–energy nutritional status. As a matter of fact, it primarily reflects renal function (e.g., early nephropathy) and has no established role in nutritional assessment or screening. Consequently, further studies using appropriate biochemical markers are needed.

Additional contributing factors may include the generally healthy condition of the participants, the absence of acute illness, and the possibility that nutritional, functional, and biochemical assessments were not performed concurrently, all of which may have reduced the likelihood of detecting significant associations.

Other important limitations of the present study, in addition to the cross-sectional design, the use of urinary rather than serum albumin, the lack of information regarding the interval between functional and biochemical assessments, and the apparently healthy condition of the participants, concern the adaptations of the methods used to assess malnutrition and frailty, as well as the insufficient data available to evaluate the presence of sarcopenia according to the EWGSOP criteria, which has long been recognized as a consequence of malnutrition. Future studies should address these limitations to provide more comprehensive insights into this topic.

## 5. Conclusions

Significant and moderate correlations were found between CC and DEXA-based ASM. This scenario impacts the prevalence of malnutrition identified according to these assessment tools, but not their association with health conditions. More studies employing more rigorous study designs (e.g., longitudinal studies) and examining the impact of serum albumin levels are necessary to provide more comprehensive insights into this topic.

## Figures and Tables

**Table 1:** Descriptive statistics of the study population (*n* = 1048).

Variable	Mean ± SD/*n* (%)
**Demographics**	
Age at Screening (years)	74.4 ± 6.3
Gender (male)	529 (50.9%)
**Anthropometrics and Body Composition**	
Body Mass Index (kg/m^2^)	27.6 ± 5.3
Maximal Calf Circumference (cm)	36.8 ± 3.9
ALM DEXA (kg)	19.5 ± 5.2
**Physical Performance**	
8-foot Walking Speed (m/s)	0.9 ± 0.3
**Clinical and Functional Measures**	
Multimorbidity (≥2 diseases)	464 (51.7%)
Albumin (urine, mg/L)	3.7 ± 1.8
Protein intake (g/kg/day)	0.89 ± 0.43
Caloric intake (kcal/day)	1693.6 ± 689.9
Frailty (%)	122 (11.6)
**Frailty criteria (%)**	
Fatigue	115 (11.0)
Resistance	37 (3.5)
Ambulation	280 (26.7)
Illness	70 (6.7)
Loss of Weight	70 (6.7)
**Disability (%)**	122 (11.6)

**Table 2:** Pearson’s correlation.

	DEXA-Based ASM	*p*-Value
Calf circumference	0.592	0.001
Calf circumference-based ASM	0.598	0.001

ASM = Appendicular skeletal muscle.

**Table 3:** Logistic regressions.

Unadjusted				Adjusted *		
Variable	OR	*p*-Value	95% CI (Lower, Upper)	OR	*p*-Value	95% CI (Lower, Upper)
Disability						
CC-based Malnutrition	1.638	0.206	0.763, 3.518	1.680	0.197	0.764, 3.969
DEXA-based Malnutrition	1.417	0.380	0.651, 3.087	1.711	0.194	0.760, 3.848
Frailty						
CC-based Malnutrition	2.908	0.001	1.832, 4.615	1.563	0.001	1.240, 1.970
DEXA-based Malnutrition	3.141	0.001	1.976, 4.994	1.699	0.001	1.306, 2.133

CC = Calf circumference; CI = Confidence interval; OR = Odds ratio. * Adjusted for age, sex, physical activity levels, and polypharmacy.

## Data Availability

The data analyzed in this study are publicly available from the National Health and Nutrition Examination Survey (NHANES) and can be accessed at https://wwwn.cdc.gov/nchs/nhanes/default.aspx (accessed on 1 September 2025).
